# Biodiversity and Evaluation of Genetic Resources of Some Coffee Trees Grown in Al-Baha, Saudi Arabia

**DOI:** 10.3390/cimb47030136

**Published:** 2025-02-20

**Authors:** Fatima Omari Alzahrani, Mohammed Obeid Alshaharni, Gamal Awad El-Shaboury, Abdelfattah Badr

**Affiliations:** 1 Department of Biology, Faculty of Sciences, Al-Baha University, Al-Baha 65729, Saudi Arabia; 2 Biology Department, College of Science, King Khalid University, Abha 61413, Saudi Arabia; maasalim@kku.edu.sa (M.O.A.); jelshaboury@kku.edu.sa (G.A.E.-S.); 3 Botany and Microbiology Department, Faculty of Science, Helwan University, Cairo 11795, Egypt

**Keywords:** biodiversity, ISSR markers, SCoT, phylogenetic, *Coffea arabica*

## Abstract

The biodiversity of 12 coffee (*Coffea arabica* L.) cultivars collected from the Al-Baha region in the southwest of Saudi Arabia was evaluated using 25 morphological variations and genetic diversity as demonstrated by molecular polymorphism generated by eight Inter Simple Sequence Repeats (ISSRs) and nine Start Codon Targeted (SCoT) primers. Substantial variations were scored in the morphological traits reflected in the clustering of the examined cultivars in PCA of the coffee cultivars. The examined cultivars were grouped in two groups, one included the cultivars coded Y5, Y6, R113, and Y7 and the other group comprised two clusters; one comprised cultivars coded R8 and R4 and the other comprised cultivars R112, R114, and Y2. In the meantime, the cultivars coded R9 and R111 were differentiated together from other cultivars, while the Y3 cultivar was confirmed by the analysis of ISSR data and SCoT data, which also support the grouping of R9 and R111 cultivars. Principle Component Analysis (PCA) of morphological, ISSR, and SCoT data as a combined set differentiated the examined species into four groups in a scatter plot in agreement with their separation in the cluster trees. The diversity profile among the examined *C. arabica* cultivars proved that R111 and R4 cultivars are highly diverse, while R8 and Y5 cultivars exhibit low diversity. Alpha diversity indices indicated that R9 and R111 cultivars are the most dominant and stable *C. arabica* cultivars among the examined samples in the study region.

## 1. Introduction

*Coffea arabica* L. belongs to the Coffea genus within the Rubiaceae family, comprising around 124 species. Only two species, *C. arabica* (*Arabian coffee* L.) and *C. canephora* Pierre (Robusta), possess commercial importance [1]. Arabian coffee originated in Ethiopia but is currently expanding globally. In Saudi Arabia, Arabian coffee is predominantly cultivated in the Al-Baha region, Asir (Hada Mountain region), and Jazan provinces (Al-Dayer Bani Malek), where trees exceeding 100 years in age are located [2]. High-quality coffee from these regions, known as Khoulani coffee, is well-known worldwide. Saudi Arabia produces some of the best coffees in the world as coffee is grown under mostly organic conditions without the use of pesticides, herbicides, and artificial fertilizers [3]. Nonetheless, the insufficiency of water resources for irrigation and the degradation of terraces may result in damage to coffee’s genetic resources, owing to climate change. Furthermore, producers have replaced coffee with other monoculture crops deemed of greater importance [4]. Khoulani and Tufahi cultivars exhibited notable responses to the impacts of drought on gas exchange, water relations, and osmotic adjustment, as studied by Tounekti et al. (2018) in four Arabica coffee cultivars grown in southwestern Saudi Arabia [3]. Genetic variation is an essential component of biodiversity, necessary for species reproduction, and it is vital for the adaptation of species to changing environments [5]. Consequently, genetic variation is essential for the development of novel types via plant breeding. Advantageous genetic traits can be included into cultivars to enhance agricultural productivity and attributes related to crop quality, disease resistance, etc. Tounekti et al. (2017) assessed the genetic diversity of Arabian coffee cultivars from southwest Saudi Arabia using physical traits and proposed four cultivars for breeding initiatives [6]. Such germplasms may demonstrate significant characteristics, including resilience to abiotic and biotic stressors. Nonetheless, the genetic basis, origins, and magnitude of this divergence remain unidentified. The germplasm gathered from the southwestern regions of Saudi Arabia, characterized by adverse conditions, may serve as a foundational material for the development of cultivars resilient to environmental challenges.

Molecular markers are currently recommended for quantifying and verifying genetic diversity at both species and cultivar levels [5,7]. Molecular characterization of wild cultivars and cultivated variants of *C. arabica* in Ethiopia revealed adequate polymorphism among the germplasm [8]. The integration of molecular marker analysis and phytochemical profiling can yield a thorough comprehension of the genetic diversity and chemical composition of *C. arabica* cultivars in Saudi Arabia [9]. This information can inform conservation initiatives, pinpoint unique characteristics for breeding programs, and perhaps facilitate the creation of distinctive Saudi Arabian coffee products with appealing flavor and aroma profiles. However, the biodiversity research on *C. arabica* in southwest Saudi Arabia has been restricted to either morphological differences [10] or polymorphisms of molecular markers [11].

Al-Ghamedi et al. (2023) demonstrated that it is vital to begin with an evaluation of genetic diversity to develop significant breeding programs for the country. Identifying and describing genotypes can improve breeding effectiveness over direct selection of desired traits and genes. The use of molecular markers can enhance crop development efforts significantly. These markers have a direct connection to the genotype’s qualities, allowing for the rapid development of new genotypes compared to traditional selection practices, particularly when measuring traits is difficult [11].

This study is crucial for establishing a foundational baseline to improve the newly established coffee city in Al-Baha, an investment initiative focused on advancing coffee farming in the area. The city comprises an area of 1,600,000 square meters. The city intends to cultivate 300,000 coffee trees and provide over 1000 employment possibilities, thus enhancing the local agricultural economy (personal communication). The development of the coffee city project is integral to Saudi Arabia’s Vision 2030, which focuses on improving local coffee production. The initiative depends on the cultivation of local coffee varieties; hence, identifying these varieties is deemed crucial for the support of this city. The choice of Al-Baha for the coffee city initiative is influenced by substantial factors such as the area’s climate compatibility and its rich history of coffee cultivation.

So, our study aims to evaluate the coffee tree biodiversity in the Al-Baha region using morphological differences and molecular markers, specifically utilizing the profiling of Inter Simple Sequence Repeats (ISSRs) and Start Codon Targeted (SCoT) markers.

## 2. Materials and Methods

### 2.1. Plant Material and Morphological Measurements

**Figure 1:**
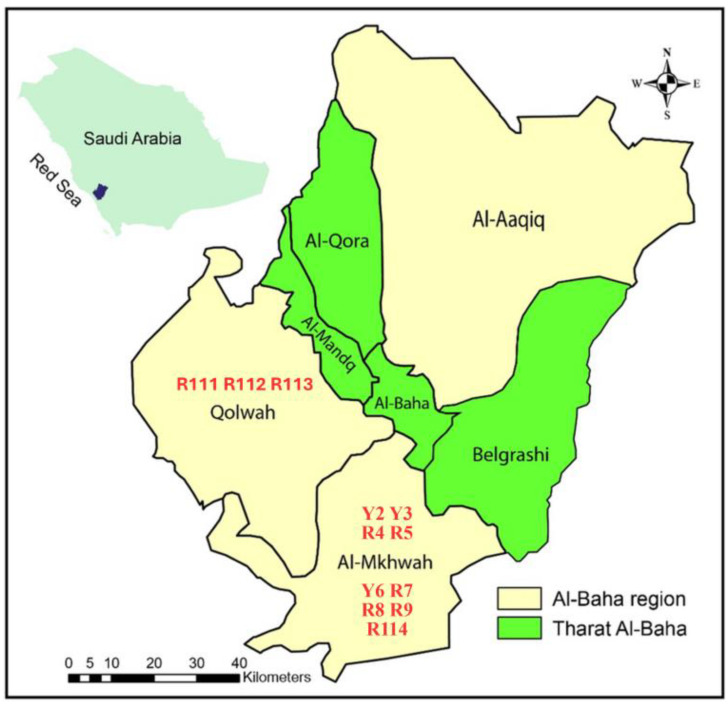
Map of Al-Baha region, southwest of Saudi Arabia, illustrating the areas and sites of collection of the 12 Arabian coffee cultivars; plotted and coded as given in Table 1.

Samples of mature flowering Arabian coffee plants were collected from 12 cultivars in two areas (five samples for each accession) from their natural habitats in the Al-Baha region, southwest of Saudi Arabia in the summer of two successive years (2022–2023). The names and sites of collection of the collected cultivars are given in Table 1 and a map of the study region is shown in Figure 1. A detailed description of 25 morphological traits for each accession including quantitative, qualitative, and presence/absence of characters was performed. The average value of every quantitative character ± standard deviation was calculated. The state of the qualitative and the present character is recorded in Table 2 based on the species description of Migahid in 1996 [12], Collenette in 1999 [13], and Chaudhary in 2001 [14]. Voucher specimens of the 12 *C. arabica* cultivars have been deposited in the Herbarium of the Biology Department, Faculty of Science, Al-Baha University, Saudi Arabia.

### 2.2. DNA Extraction and ISSR Fingerprinting

DNA was extracted and purified from the young leaves of mature plants of the collected samples of the Arabian coffee plants representing all the collected cultivars using Qiagen DNeasy™ Plant Minikit following the manufacturer’s protocol (Qiagen Inc, Valencia, CA, USA). Eight ISSR and nine SCoT primers were used for DNA fingerprinting. The selection of ISSR and SCoT primers was guided by their established effectiveness in earlier genetic diversity investigations. These primers are known for their capacity to generate polymorphic and reproducible bands, making them particularly suitable for analyzing the coffee genome, as evidenced by previous studies [15,16,17].

**Figure 2:**
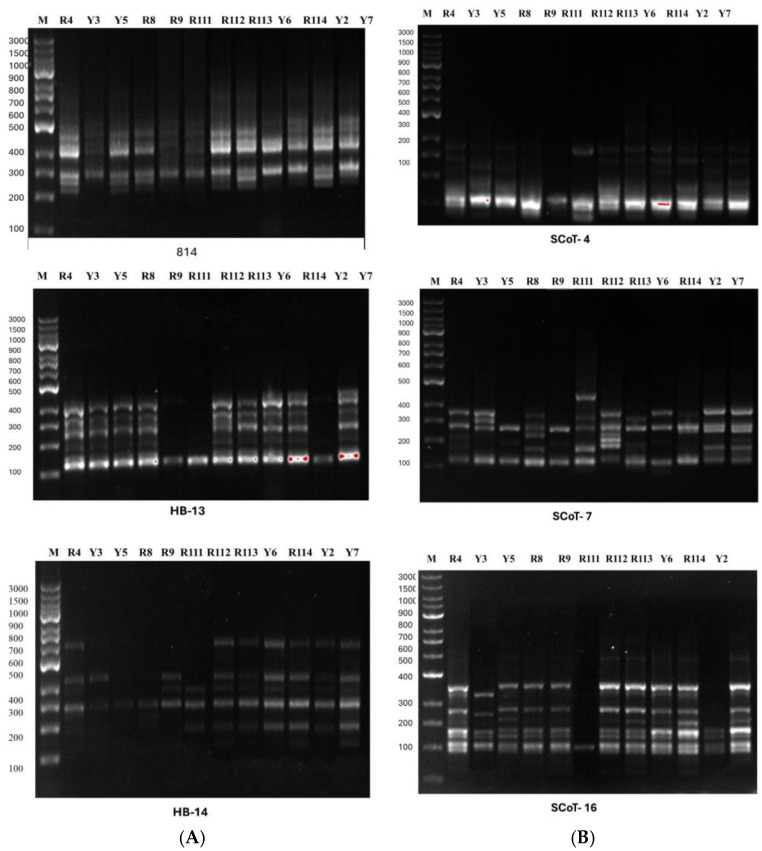
Photographs illustrating (**A**): ISSR and (**B**): SCoT fingerprinting profiles produced by three primers for *C. arabica* cultivars as coded in Table 1. M: 100 bp marker DNA ladder. Cultivar codes as given in Table 1.

The name, sequence, number of polymorphic bands, and percentage of polymorphism of the 17 primers are given in the online Supplementary Table S1. In the amplification reactions of genomic DNA, a total of 25 µL reaction mix was prepared (12.5 µL Thermo Scientific Maxima Hot Start PCR Master Mix (2×, 0.5 µL primer, 0.5 µL template DNA and 11.5 µL nuclease-free water-R0581). Amplification conditions were improved using a gradient Biometra Uno thermal cycler, Germany. In total, 20 µL of the PCR products of each primer and 2 µL of loading buffer were mixed and loaded into the wells of 1.7% agarose gel. The DNA fingerprinting of the studied primers was visualized and photographed using a Gel Works 1D advanced gel documentation system (UVP, Cambridge, UK). Figure 2 shows the ISSR and SCoT fingerprinting profile produced by three of each ISSR and SCoT primers for the examined Arabian coffee cultivars. For data analysis, each ISSR and SCoT band was considered a single locus and scored as 1 for presence and 0 for absence (ISSR and SCoT fingerprinting scoring is given in the online Supplementary Table S2).

### 2.3. Data Analysis

The morphological traits were assigned codes from 0 to 3 for data analysis. The descriptor states and codes for data analysis of the examined morphological traits can be found in the online Supplementary Table S3. Leaf length and width were recorded using a leaf area meter (CI-202 Portable Laser Leaf Area Meter). To determine the coffee fruits’ fresh weight, the fruits were first cleaned and dried using blotting papers. The weight of the fruits was then measured using an electronic balance. The fruits and seeds were left to dry in an oven set at 70 °C for three days. Once completely dry, the weight of the dry fruits and seeds was measured using an electronic balance.

The relationships between Arabian coffee cultivars were evaluated separately and together based on variations in the morphological traits and molecular fingerprinting polymorphism. Two software programs were used for data analysis; the NTSYS-pc software version 2.2. [18] was utilized to construct trees showing the relationships and to calculate the similarity level among the examined Arabian coffee cultivars using a simple matching coefficient [19]. Clustering of the examined cultivars was also carried out based on squared Euclidean distance to create a distance tree using PAST-pc Version 4.11 [20]. Principal Component Analysis (PCA) was used to build a scatter diagram of the examined cultivars and diversity profile curve, and alpha diversity plots were generated using the PAST-pc using Shannon diversity index [21]. It is important to note that PCA is sensitive to the relative scaling of the original variables in the scatter plot visualization [20]. Alpha diversity means the diversity of cultivars within a specific local area, indicating species richness in a particular community. For instance, alpha diversity measures the observed species diversity within a defined plot or ecological unit, such as a field, a pond, or a rainforest [22]. All morphological measurements were conducted three times independently, and the results are shown as the average value ± standard deviation (SD). A one-way analysis of variance (ANOVA) was applied to the data using Statistica 7.1.

### 2.4. iMEC Analysis

According to Amiryousefi et al. [23], the Online Marker Efficiency Calculator (iMEC software) is an easy-to-use program that computes fundamental polymorphism indices for individual markers, including the heterozygosity index (H), polymorphism information content (PIC), discriminating power (D), effective multiplex ratio (E), marker index (MI), arithmetic mean heterozygosity (Havp), and resolving power (R) [23]. The iMEC application is obtainable at https://irscope.shinyapps.io/iMEC/, accessed on 17 February 2025. The multiplex ratio (MR) = total bands/total primers used = 132/17 = 7.76. The iMEC analysis involved the assessment of 17 molecular markers, comprising 8 ISSR and 9 SCoT primers in 12 Arabian coffee cultivars using the online iMEC software application. The results of the calculations are summarized in Table 3. The effectiveness of the primers in distinguishing between different collected cultivars was assessed based on the D parameter (discriminating power of primer), as described by Ahmed et al. (2019) [24].

## 3. Results

### 3.1. Morphological Variation Among Coffea arabica Cultivars

Twenty-five morphological traits were utilized for the 12 collected *C. arabica* cultivars (Table 2). The morphological qualitative characteristics exhibited significant variance among the analyzed cultivars, particularly in all assessed attributes, with the exception of a cherry color, which was consistently light red across all tested cultivars, except for the Y7 accession, which displayed a tint of orange. All investigated cultivars had a lanceolate leaf shape, except for cultivars R111, R112, and R114, which displayed an ovate leaf shape, while R113 exhibited an elliptic shape. The quantitative characteristics indicate that cultivars from high-elevation sites with mild temperatures typically exhibit higher plant sizes compared to those from lower elevations in arid regions. For instance, the plant height of samples obtained from elevated regions is much greater; specifically, R4, Y3, Y5, R8, Y6, Y2, and Y7, collected from the Al-Mehkwa region at an elevation of 1684 m asl, exhibited a range from long to moderate plant height (Table 1 and Table 2). The short cultivars, such as R9 and R114, were gathered from the Al-Mehkwa region at lower elevations of 1104 m and 1107 m above sea level, respectively. Despite the R111 accession having considerable heights, it was obtained from an intermediate elevation of 1321 m above sea level in the Qalwa region. Each gathered accession possesses distinct characteristics derived from our observations and insights provided by experienced growers: R4, Y5, and R111 exhibit high-quality cherries; Y3 demonstrates early yield; R8 is noted for great cherry output; and R9 and R114 are recognized for both high yield and good quality cherries. R112 exhibits robust vegetative growth, R113 and Y2 demonstrate commendable growth and yield, Y6 trees exhibit tolerance to water scarcity, and Y7 produces large cherries.

### 3.2. Diversity of Cultivars Based on Morphological Variations

**Figure 3:**
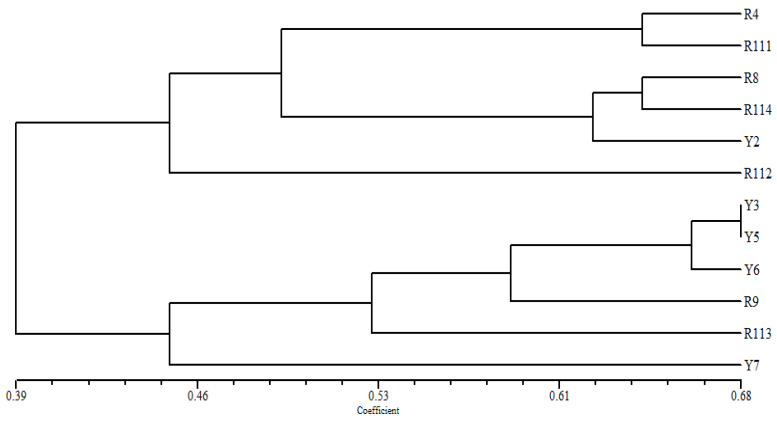
UPGMA and NTSYS distance tree, based on the analysis of morphological traits, computed with the SM coefficient, showing the relationships among the examined Arabica coffee cultivars.

Based on the simple matching coefficient among the studied *C. arabica* cultivars, the UPGMA-NTSYS-pc cluster tree (Figure 3) separated the examined cultivars into two main groups. The first group compressed two clusters, the R4 accession clustered with R111. However, R8, R114, and Y2 were found in the other cluster. In contrast, R112 delaminated from this group at a very low similarity level. The other group comprised Y3 and Y5 at the highest similarity level with the Y6 and R9 accessions in one cluster. The R113 and Y7 cultivars were separated individually from this group.

### 3.3. ISSR and SCoT Fingerprinting Polymorphism in Coffea arabica Cultivars

The ISSR and SCoT fingerprinting profiles produced 132 bands (markers); 95 are polymorphic, 16 are monomorphic, and 21 are unique. The highest number of bands (12) was produced by primer SCoT-14 and the lowest number (3) was produced by primer HB-8. The percentage of polymorphism of all primers was calculated and is given in Online Supplementary Table S1. Data generated by the ISSR and SCoT fingerprinting of the 12 *Coffea arabica* cultivars are scored in Online Supplementary Table S2.

The polymorphism indices for individual primers were determined using iMEC software as basic metrics. More detailed information about the fundamental measurement of polymorphism indices for primers is provided in Table 3. The average heterozygosity index (H) was found to be 0.495. Additionally, the polymorphism information content (PIC) for each primer averaged 0.373. The primer SCoT-16 had an effective multiplex ratio (E) of 5.417, while primer HB-8 had a ratio of 2.5, with an average ratio of 4.754. The primer resolving power (R) ranged from 1.00 (HB-8) to 6.00 (SCoT-7) with an average of 4.13. The arithmetic means H (Havp) varied from 0.003 with primer SCoT-14 to 0.008 with primer HB-8 with an average of 0.004. Primer SCoT-13 showed the lowest value for marker index (MI) at 0.01, while primer HB-10 had a value of 0.026 with an average of 0.023. The primer discriminating power (D) ranged from 0.310 for primer HB-8 to 0.914 for SCoT-13, with an average of 0.777 (Table 3).

### 3.4. Diversity of Coffea arabica Cultivars Based on ISSR Markers

**Figure 4:**
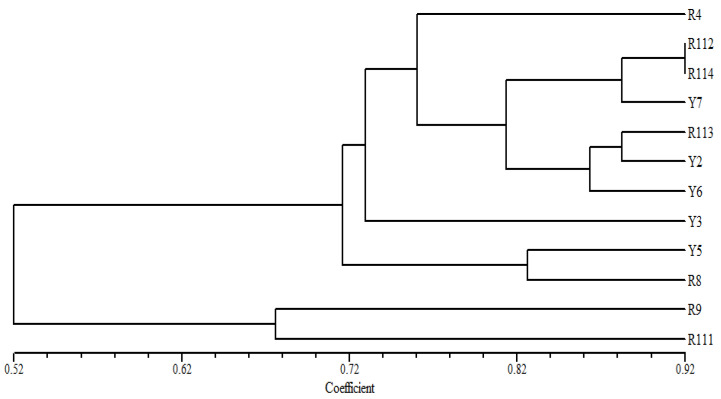
UPGMA distance tree computed using NTSYS-pc version 2.2, based on the analysis of ISSR data, showing the relationships among the examined *C. arabica* cultivars in the study area.

In the cluster tree, based on the ISSR fingerprinting analysis (Figure 4), the examined *Coffea arabica* cultivars are divided into two groups; a small group of the R9 and R111 cultivars is clearly differentiated, as two separate identities can be found at a relatively low similarity level from the other examined cultivars. In the second main group, the cultivars Y5 and Y8 are clustered at a relatively high similarity, while R4 and Y3 are delaminated individually in this group. The remaining cultivars R112 and R114 are found at the highest similarity level in one cluster with the Y7. Likewise, the R113 and Y2 cultivars are grouped in one cluster with the Y6 cultivar.

### 3.5. Diversity of the Arabian Coffee Cultivars Based on SCoT Markers

**Figure 5:**
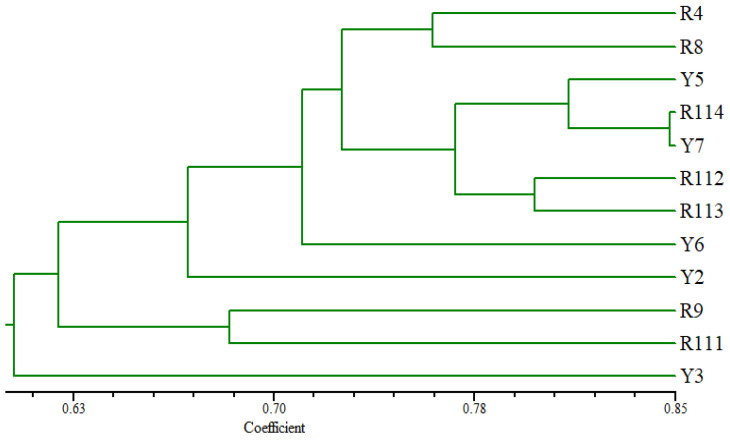
UPGMA distance tree computed using NTSYS-pc version 2.2, showing the relationships among the examined *C. arabica* cultivars based on the analysis of SCoT data.

The tree based on the SCoT fingerprinting analysis (Figure 5) revealed that the Arabian coffee cultivars are grouped into three main groups, where the Y3 cultivar was differentiated as a separate identity at the lowest similarity level from the examined cultivars. R9 and R111 separated at a relatively low similarity level in one group. The second group consisted of two clusters, where R114 and Y7 cultivars clustered at a high similarity level with Y5 accession; the second cluster contained R112 and R113 at a relatively low similarity level. R4 and R8 cultivars were clustered in the other group. The Y6 and Y2 cultivars were distinguished individually from the other examined cultivars.

### 3.6. Relationships of Arabian Coffee Cultivars Based on Morphological Variations Combined with ISSR and SCoT Markers

**Figure 6:**
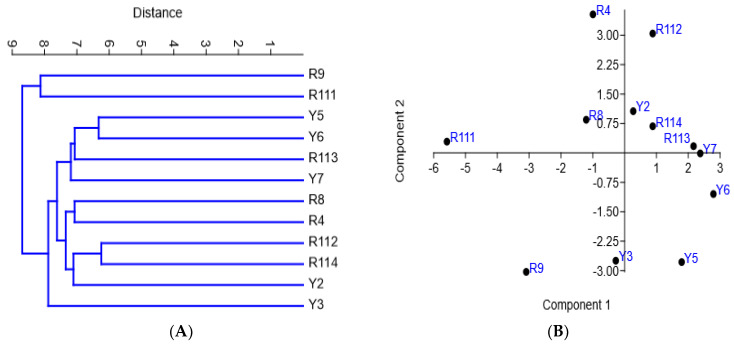
UPGMA distance tree (**A**) and PCA scatter diagram (**B**) of the examined Arabian coffee cultivars, constructed using the PAST-pc software version 4.13, showing the relationships among cultivars based on the analysis of variation in the morphological traits and ISSR–SCoT fingerprinting polymorphism.

**Figure 7:**
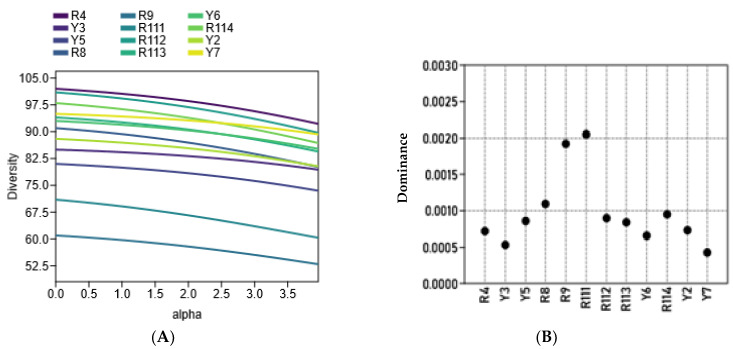
(**A**): The diversity profile and (**B**): alpha diversity, which were calculated using the Pasta pc program version 4.13, among the twelve studied *C. arabica* cultivars.

The diversity of the examined cultivars based on morphological variations and molecular markers’ (ISSR and SCoT) polymorphism was assessed using clustering analysis, based on the Euclidean equation using the PAST software version 4.13 (Figure 6A) and a PCA scatter plot (Figure 6B). The cluster tree confirmed the separation of R9 and R111 cultivars as separate identities in one group. The cluster tree indicated more differentiation between the rest of the examined cultivars where Y5 and Y6 were clustered together, and the R4 and R8 cultivars and R112 and R114 cultivars were also clustered together. The examined cultivars are clearly differentiated into four groups by the PCA scatter plot (Figure 6B), which agrees mostly with their separation in the cluster tree. The four groups are 1. R4, R8, and R111; 2. R112, R113, R114, and Y2; 3. Y5, Y6, and Y7; and 4. R9, and Y3. Principal Component Analysis (PCA) results among the examined coffee arabica accessions are shown in in Online Supplementary Table S4. Figure 7A shows that the R4, R9, and Y6 cultivars were highly diverse *C. arabica* cultivars, while the R8 and Y5 cultivars exhibited low diversity. The alpha diversity indices (Figure 7B) indicated that the R9 and R111 cultivars were the most dominant and stable *C. arabica* cultivars among the examined samples. Additionally, the analysis revealed that the Y3 and Y7 cultivars were the least dominant *C. arabica* cultivars.

## 4. Discussion

Genetic diversity, both within and between plant cultivars, in natural settings may be influenced by changes in environmental conditions. Cultivars with elevated genetic diversity have greater resilience to habitat degradation and environmental alterations [25]. The assessment and measurement of genetic diversity and its distribution throughout time and space can be facilitated by studies on the species’ genetics [26]. Genetic variability can be assessed using phenotypic and molecular levels of analysis [27]; however, recently, molecular markers have become more widely accessible for use in molecular taxonomy, cultivar identification, and marker-assisted selection in plants due of developments in DNA fingerprinting techniques [28,29].

The analysis of the studied morphological traits revealed significant variation among the Arabica coffee cultivars for most of the morphological traits. Part of the variation was likely due to the influence of environmental factors such as rainfall, soil fertility, differences in agricultural practices, and the altitude of the collection site from sea level. Most qualitative traits such as plant shape, growth habit, leaf petiole color, and leaf and fruit shape vary between the examined cultivars. For example, the plant shape was pyramidal in four cultivars (R4, R8, R111, and R114) but ellipsoid in the Y5, R9, R112, Y6, and Y2 cultivars, and it was conical in the two cultivars R113 and Y7 only (Table 2). This may be correlated with the wide morphological variation in Arabica coffee species in different geographical regions under different environmental conditions [10]. Phenotypic variation in some morphological characters, like plant heights, could be related to variations in the elevation and landscape topography of the area in which the plants grow.

As for the quantitative characteristics in our study, the cultivars collected from sites at high elevations and moderate temperatures generally have larger plant sizes, canopy diameter, leaf width, and leaf petiole length than cultivars at lower elevations in more arid areas. The Arabica coffee productivity is also affected by the site of collection, where samples collected at high elevations have a higher weight for 100 fruit fresh and dry weight than those collected at lower elevations (Table 2). The cluster tree generated based on a simple matching coefficient among the studied Arabica coffee cultivars separated the R112 and Y7 cultivars from the other cultivars, indicating that these two cultivars have unique morphometric characteristics. Based on our observations and information shared by Arabica coffee growers in study areas, the Y7 cultivar has a large cherry and R112 has good vegetation growth. Previous studies using morphometric traits reported that it is often difficult to differentiate Arabica coffee cultivars based only on a limited number of traits [30,31]. In the current study, 25 traits related to tree canopy structure, fruit and seed morphology, mass and color, leaf properties, and internode length were used to identify the cultivars and provided more information on the morphological variation among the examined cultivars, but they indicated a low level of genetic differentiation of cultivars based on their geographic distribution in the study area.

The polymorphic information content (PIC) provides an estimate of a locus’ discriminatory power by considering the number and relative frequencies of alleles [24,25]. The PIC values range from 0 to 1 and indicate the probability of detecting a polymorphism between individuals. The current study used 17 different ISSR and SCoT marker combinations to determine the genetic diversity among 12 local Arabica coffee cultivars. The 17 primers produced 95 polymorphic bands with a high-level polymorphism percentage, averaging 85.83% in the 12 Arabica coffee cultivars (Table 3 and Table S1), where a high percentage of polymorphism indicates a high level of genetic diversity within the population or species. Our results agree with Yunita et al. (2020), who reported comparable findings in their study of genetic diversity in Arabica coffee in Indonesia, utilizing 16 combinations of molecular markers [22]. The average number of polymorphic loci was found to range from 19 to 23, while the information on polymorphic loci varied from 82.6% to 100% with an average of 95%. The MR was 7.76 bands per primer, EMR was 4.754, MI was 0.023, PIC was 0.373 and the average RP of all studied primer combinations was 4.13 (Table 3). Using genetic markers, Kumar et al. (2019) recorded comparable findings for a Jojoba cultivar (EMR = 5.36, PIC = 0.47, RP = 8.07 and I = 2.59) [31]. A higher value of MI, which assesses the performance of a molecular marker system, shows that the molecular markers are reliable in identifying genetic variations among several cultivars [32].

The cluster tree generated for the examined Arabica coffee cultivars based on the analysis of ISSR data (Figure 4) grouped the 12 cultivars into three groups. The R9 and R11 cultivars clustered in one group, representing the most divergent cultivars, collected from Al-Mehkwa and Qalwa, respectively. The second group comprised Y5 and R8 cultivars collected from the Al-Mehkwa region but from different GPS locations. The R112 and R114 cultivars clustered at a high similarity level, which were also collected from Qalwa and Al-Mehkwa, respectively. However, the results indicate that the cultivars of the Arabica coffee harvested from the same area were grouped into different clusters except for the Y5 and Y6 cultivars, which were found in the same cluster based on the combined analysis of morphological variations and the molecular polymorphism of the ISSR and SCoT markers (Table 1), indicating substantial genetic diversity within the cultivars of each region. This is not consistent with the results of Yunita et al. (2020), who reported that molecular data allowed for the grouping of the Arabica cultivars into three clusters correlated with their geographic distribution [22]. However, other studies have suggested a strong influence of geographic origin on diversity among the Arabica coffee cultivars [33,34].

The clustering of the studied Arabica coffee cultivars, based on the analysis of the SCoT fingerprinting profile, resulted in a grouping of the 12 cultivars that shows substantial differences compared to their groupings based on the analysis of ISSR data (Figure 5). However, the clustering of R9 and R111 cultivars together agrees with the grouping based on ISSR data analysis. These results indicate that these two cultivars share unique characteristics that distinguish them from the other studied cultivars. They are characterized by good vegetation growth and large cherry size. The R9 accession is also characterized by high productivity (Table 2). Figure 5 also illustrates that the Y3, Y2, and Y6 cultivars were distinctly separated from the other cultivars, confirming a high level of biodiversity among the examined cultivars. This is supported by the grouping of R4 and R8. The PCA scatter plot, generated using the Euclidean coefficient based on the analysis of morphological trait variations, ISSR, and SCoT fingerprinting polymorphism (Figure 6B), confirmed the grouping of the studied Arabica coffee cultivars and confirmed that the cultivars have high genetic diversity.

The diversity profile and alpha diversity analysis of the examined cultivars are depicted in Figure 7A,B. Alpha diversity pertains to the diversity on a local scale and describes the species diversity within a community, such as within a defined plot or ecological unit like a pond, field, or patch of forest [22]. In the current study, the diversity profile and alpha diversity among the examined Arabian coffee cultivars proved that the R4, R9, and Y6 cultivars are highly diverse, while the R8 and Y5 cultivars exhibit low diversity (Figure 7A). The alpha diversity indices (Figure 7B) indicate that the R9 and R111 cultivars are the most dominant and stable Arabian coffee cultivars among the examined cultivars. Characters exhibiting significant diversity are expected to facilitate substantial gene transfer in breeding programs [35]. In plants, alpha diversity is frequently associated with the number of species recorded during the assessment of a vegetation plot of specified dimensions [36]. Alpha diversity can be used to compare genetic diversity between different populations of the same species. This helps identify populations that are genetically depauperate and may require conservation interventions. For example, a population with higher alpha diversity might be prioritized as a source for genetic rescue efforts to bolster diversity in other populations [21]. A diversity profile illustrates a curve that represents the associated values of a substantial array of diversity indices. Thus, the profile portrays the views of diversity from many different vantage points simultaneously [37]. Ovalle-Rivera et al. (2015) suggested that Arabian coffee growers might face numerous difficulties as environmental conditions could become increasingly unfavorable for Arabica coffee production in the main producing nations because of climate change [38]. This is particularly applicable to the southwest Arabian Peninsula region and the Middle East [11]. The current study suggests that the local Arabian coffee cultivars have relatively diverse genetic traits and have evolved in mostly semi-arid environments and are frequently influenced by drought. This shows that these genetic resources may hold valuable genes that confer resistance to abiotic stress [3]. It is recommended to preserve and utilize these genetic resources in future coffee breeding programs to improve the coffee crop’s resilience to environmental challenges.

Previous research has confirmed substantial genetic variation across Arabica coffee cultivars in Saudi Arabian and Yemeni varietals [11,39,40,41]. It was documented that most Yemeni coffee varieties originated from ancient “heirloom” cultivars of *C. arabica* that were initially naturalized centuries ago [41,42]. The findings of the current study and several others addressing genetic diversity in Arabica coffee cultivars in Saudi Arabia and Yemen bolster the proposition that the Arabian Peninsula constitutes the most significant hub of coffee diversity beyond the species’ original center in Ethiopia and South Sudan [6,41,42].

## 5. Conclusions

The biodiversity of 12 Arabian coffee cultivars from Al-Baha region was evaluated using morphological variations in 25 morphological traits and the molecular polymorphism generated by eight ISSR and nine SCoT primers. The PCA of morphological, ISSR data, and SCoT data differentiated the examined species into four groups in a scatter plot. The differentiation of the cultivars in the PCA scatter diagram agrees with their separation in the cluster trees. The diversity profile among the examined Arabian coffee cultivars indicated that the R4, R9, and Y6 cultivars are highly diverse, while the R8 and Y5 cultivars have low diversity. Alpha diversity indices indicated that the R9 and R111 cultivars are the most dominant and stable Arabian coffee cultivars. Preserving this material and incorporating it into breeding programs is likely to increase the genetic diversity of coffee in the future, helping the crop to better withstand environmental challenges. Additionally, molecular and taxonomical studies may reveal new varieties or subvarieties of Arabian coffee arabica in the Al-Baha region. The molecular biodiversity of coffee is a critical resource for ensuring the resilience, quality, and sustainability of coffee production. It offers significant potential for scientific research, economic development, and cultural preservation, making it essential for the future of coffee and the broader food industry.

## Figures and Tables

**Table 1:** The 12 cultivars of the Arabian coffee plants collected from three areas in the Al-Baha region, as well as GPS location and elevation of sites from which the cultivars were collected.

No.	Cultivars Code	Area	GPS Location		Elevation (m) Asl
1	R4	Al-Mehkwa	19°50′32.002″ N	41°18′29.08″ E	1685
2	Y3	Al-Mehkwa	19°50′9.74″ N	41°18′49.038″ E	1684
3	Y5	Al-Mehkwa	19°50′9.74″ N	41°18′49.038″ E	1684
4	R8	Al-Mehkwa	19°50′32.002″ N	41°18′29.08″ E	1684
5	R9	Al-Mehkwa	19°44′44.623″ N	41°21′19.939″ E	1104
6	R111	Qalwa	19°55′40.242″ N	41°9′15.18″ E	1321
7	R112	Qalwa	19°55′40.242″ N	41°9′15.18″ E	1321
8	R113	Qalwa	19°55′41.242″ N	41°9′18.18″ E	1351
9	Y6	Al-Mehkwa	19°50′9.74″ N	41°18′49.038″ E	1684
10	R114	Al-Mehkwa	19°50′32.91″ N	41°18′28.693″ E	1107
11	Y2	Al-Mehkwa	19°50′9.74″ N	41°18′44.068″ E	1684
12	Y7	Al-Mehkwa	19°50′19.74″ N	41°18′39.038″ E	1684

**Table 2:** A list of morphological traits and the measurements of quantitative traits and state of qualitative traits of the Arabian coffee plants in the 12 cultivars, coded as given in Table 1.

	No.	Character	Arabian Coffee Cultivars					
			R4	Y3	Y5	R8	R9	R111
Qualitative Characters	1	Plant Shape	Pyramidal	Ellipsoid	Ellipsoid	Pyramidal	Ellipsoid	Pyramidal
	2	Growth Habit	Tree	Shrub	Shrub	Tree	Shrub	Tree
	3	Young Leaf Anthocyanin Coloration	Week or absent	Strong	Strong	Strong	Strong	Strong
	4	Leaf Petiole Color	Green	Dark Brown	Dark Brown	Green	Dark Brown	Green
	5	Inflorescence Position	Axillary	Terminal	Terminal	Axillary	Terminal	Axillary
	6	Inflorescence on Old Wood	Absent	Present	Present	Absent	Present	Absent
	7	Fruit adherence to branch	Strong	Strong	Strong	Strong	Weak	Weak
	8	Cherry Color	Light red	Light red	Light red	Light red	Light red	Light red
	9	Leaf Shape	lanceolate	Lanceolate	lanceolate	lanceolate	lanceolate	Ovate
	10	Fruit Shape	Circular	Elliptic	Elliptic	Circular	Elliptic	Circular
Quantitative Characters	11	Plant Height	Long	Long	Long	Intermediate	Short	Long
	12	Canopy Diameter	Narrow	Wide	Wide	Wide	Intermediate	Wide
	13	Internode Length on Primary Branch	Intermediate	Short	Short	Short	Short	Short
	14	Leaf Length	Short	Short	Intermediate	Intermediate	Short	Short
	15	Leaf Width	Intermediate	Intermediate	Narrow	Intermediate	Intermediate	Narrow
	16	Leaf Margin Undulation	Intermediate	Strong	Intermediate	Week or absent	Intermediate	Strong
	17	Leaf Petiole Length cm	4.5 ± 0.24	4.6 ± 0.32	6.7 ± 0.02	5.7 ± 0.44	4.8 ± 0.29	4.4 ± 0.22
	18	No. of Flowers	25	18	14	19	18	22
	19	No. of Flower Fascicles	11	8	7	10	8	11
	20	Fruit Size cm	1.5 ± 0.69	1.5 ± 0.60	1.5 ± 0.54	1.9 ± 0.93	1.8 ± 0.19	0.8 ± 0.32
	21	Fruit Thickness	Intermediate	Intermediate	Intermediate	Thin	Intermediate	Intermediate
	22	Seed width	Intermediate	Intermediate	Intermediate	Intermediate	Broad	Intermediate
	23	100 Fruit fresh weight	163 ± 4.28	147 ± 5.24	112 ± 2.39	258 ± 9.73	256 ± 7.09	176 ± 4.51
	24	100 Fruit dry weight	49.7 ± 1.74	44.6 ± 0.24	56 ± 4.33	45.2 ± 5.64	46.9 ± 3.23	40 ± 3.63
	25	100Seed dry weight	27 ± 0.93	22 ± 0.24	24 ± 1.04	35 ± 1.35	38 ± 2.53	30 ± 6.37
Qualitative Characters	1	Plant Shape	Ellipsoid	Conical	Ellipsoid	Pyramidal	Ellipsoid	Conical
	2	Growth Habit	Tree	Shrub	Shrub	Tree	Tree	Shrub
	3	Young Leaf Anthocyanin Coloration	Week or absent	Strong	Strong	Week or absent	Strong	Week or absent
	4	Leaf Petiole Color	Dark Brown	Green	Dark Brown	Green	Green	Dark Brown
	5	Inflorescence Position	Axillary	Terminal	Axillary	Terminal	Terminal	Axillary
	6	Inflorescence on Old Wood	Present	Absent	Present	Absent	Absent	Present
	7	Fruit adherence to branch	Weak	Strong	Strong	Strong	Strong	Weak
	8	Cherry Color	Light red	Light red	Light red	Light red	Light red	Orang
	9	Leaf Shape	Ovate	Elliptic	lanceolate	Ovate	Lanceolate	lanceolate
	10	Fruit Shape	Circular	Elliptic	Circular	Elliptic	Elliptic	Circular
Quantitative Characters	11	Plant Height	Short	Short	Intermediate	Short	Long	Intermediate
	12	Canopy Diameter	Wide	Narrow	Intermediate	Wide	Intermediate	Narrow
	13	Internode Length on Primary Branch	Long	Short	Short	Short	Short	Short
	14	Leaf Length	Intermediate	Long	Intermediate	Short	Intermediate	Short
	15	Leaf Width	Intermediate	Wide	Intermediate	Intermediate	Intermediate	Narrow
	16	Leaf Margin Undulation	Strong	Strong	Strong	Strong	Strong	Intermediate
	17	Leaf Petiole Length cm	3.7 ± 1.29	4.1 ± 1.82	6.1 ± 1.22	4.3 ± 1.04	5.1 ± 1.39	4.9 ± 1.27
	18	No. of Flowers/plant	19	17	13	18	18	14
	19	No. of Flower Fascicles	10	8	6	7	8	6
	20	Fruit Size cm	1.5 ± 0.69	1.4 ± 0.60	1.4 ± 0.54	1.9 ± 0.93	1.8 ± 0.19	1.4 ± 0.32
	21	Fruit Thickness	Thin	Intermediate	Intermediate	Thin	Intermediate	Thick
	22	Seed width	Intermediate	Intermediate	Broad	Intermediate	Intermediate	Narrow
	23	100 Fruit fresh weight	133 ± 6.73	166 ± 4.266	175 ± 4.22	176 ± 6.56	178 ± 3.83	180 ± 6.89
	24	100 Fruit dry weight	44.9 ± 4.73	47 ± 2.53	49 ± 2.98	45.2 ± 3.39	48 ± 4.57	53 ± 4.34
	25	100 Seed dry weight	34 ± 1.23	24 ± 2.25	24 ± 2.27	34 ± 1.83	26 ± 1.32	23 ± 1.23

**Table 3:** Polymorphism statistics were estimated using the iMEC tool for 8 ISSR and 9 SCoT primer types using the data set from the 12 Coffea cultivars.

No	Primer Code	Total Number of Bands	H	PIC	E	Rp	H. av	MI	D
1	HB-8	3	0.278	0.457	2.500	1.000	0.008	0.019	0.310
2	HB-9	10	0.455	0.392	3.500	3.667	0.004	0.013	0.879
3	HB-10	8	0.422	0.406	5.583	3.167	0.004	0.026	0.515
4	HB-12	7	0.459	0.390	4.500	3.000	0.005	0.025	0.590
5	HB-13	6	0.389	0.420	4.417	2.167	0.005	0.024	0.461
6	HB-14	6	0.453	0.393	3.917	3.167	0.006	0.025	0.577
7	814	7	0.408	0.412	5.000	2.333	0.005	0.024	0.492
8	826	6	0.401	0.415	4.333	3.000	0.006	0.024	0.481
9	ScoT3	8	0.437	0.400	2.583	4.500	0.005	0.012	0.898
10	ScoT4	6	0.444	0.397	4.000	3.000	0.006	0.025	0.559
11	ScoT7	10	0.495	0.373	4.500	6.000	0.004	0.019	0.800
12	ScoT8	9	0.494	0.373	5.000	2.333	0.005	0.023	0.694
13	ScoT9	6	0.375	0.425	4.500	2.000	0.005	0.023	0.440
14	ScoT13	9	0.417	0.408	2.667	3.667	0.004	0.010	0.914
15	ScoT14	12	0.490	0.375	5.167	5.000	0.003	0.018	0.816
16	ScoT15	10	0.499	0.371	4.750	3.833	0.004	0.020	0.776
17	ScoT16	9	0.479	0.381	5.417	3.500	0.004	0.024	0.640
Average			0.495	0.373	4.754	4.130	0.004	0.023	0.777

Note: heterozygosity index (H), polymorphic information content (PIC), effective multiplex ratio (E), arithmetic mean of H (H. av), marker index (MI), discriminating power (D), resolving power (Rp).

## Data Availability

All the data in this study are available upon request.

## Supplementary Materials

The following supporting information can be downloaded at: https://www.mdpi.com/article/10.3390/cimb47030136/s1.
