# Supplementary material for: Biodiversity and Evaluation of Genetic Resources of Some Coffee Trees Grown in Al-Baha, Saudi Arabia
Source: Curr Issues Mol Biol. 2025 Feb 20;47(3):136. doi: 10.3390/cimb47030136 (PMC11941560; doi:10.3390/cimb47030136)
Supplement: Supplementary file 1 [file cimb-47-00136-s001.zip › cimb-3466192-supplementary.pdf]

Table S1. The number of polymorphic bands and percentage of polymorphism in the ISSR and SCoT profile of 17 primers in the collected *Coffea arabica* populations genome.

S.	Primer Code	Sequence (5'→3')	Number of Poly-Bands	Number of Uni-Bands	Number of Mono-Bands	Total Number of Bands	Polymorphism Percentage (%)
1	HB-8	$(GA)_6GG$	2	0	1	3	66.6
2	HB-9	$(GT)_6GG$	7	3	0	10	100
3	HB-10	$(GA)_6CC$	6	1	1	8	87.5
4	HB-12	$(CAC)_3GC$	4	1	2	7	71.42
5	HB-13	$(GAG)_3GC$	5	0	1	6	83.33
6	HB-14	$(CTC)_3GC$	5	0	1	6	83.33

7	814	$(CT)_8TG$	5	0	2	7	71.42
8	826	$(AC)_8C$	5	0	1	6	83.33
9	ScoT3	CAATGGCTACCACTAACG	6	2	0	8	100
10	ScoT4	CAACAATGGCTACCACCG	6	0	0	6	100
11	ScoT7	CAATGGCTACCATTAGCC	8	1	1	10	90
12	ScoT8	ACCATGGCTACCAGCGGC	4	3	2	9	77.77
13	ScoT9	ACAATGGCTACCACTGCC	4	0	2	6	66.66
14	ScoT13	ACAATGGCTACCACCGAC	4	4	1	9	88.88
15	ScoT14	CCATGGCTACCACTACCG	9	3	0	12	100
16	ScoT15	ACAATGGCTACCACCAGC	8	2	0	10	100
17	ScoT16	CCATGGCTACCACTACCC	7	1	1	9	88.88
Total			95	21	16	132	-

Samples:

1	2	3	4	5	6	7	8	9	10	11	12	13
ladder	Y2	Y3	R4	R5	Y6	R7	R8	R9	R111	R112	R113	R114

Table S2. Different *Coffee* cultivars/populations.

Primer Name: HB-8.

Band No	bp	Table S2 continued *Coffee* cultivars/populations											
		2	3	4	5	6	7	8	9	10	11	12	13	**Band type**
1	350	1	1	1	1	1	1	1	1	1	1	1	1	M
2	450	1	1	1	1	0	1	1	1	1	1	1	1	P
3	625	1	1	0	0	0	1	1	1	0	1	0	1	P
**Tot. No. of bands**		**3**	**3**	**2**	**2**	**1**	**3**	**3**	**3**	**2**	**3**	**2**	**3**	

Image:

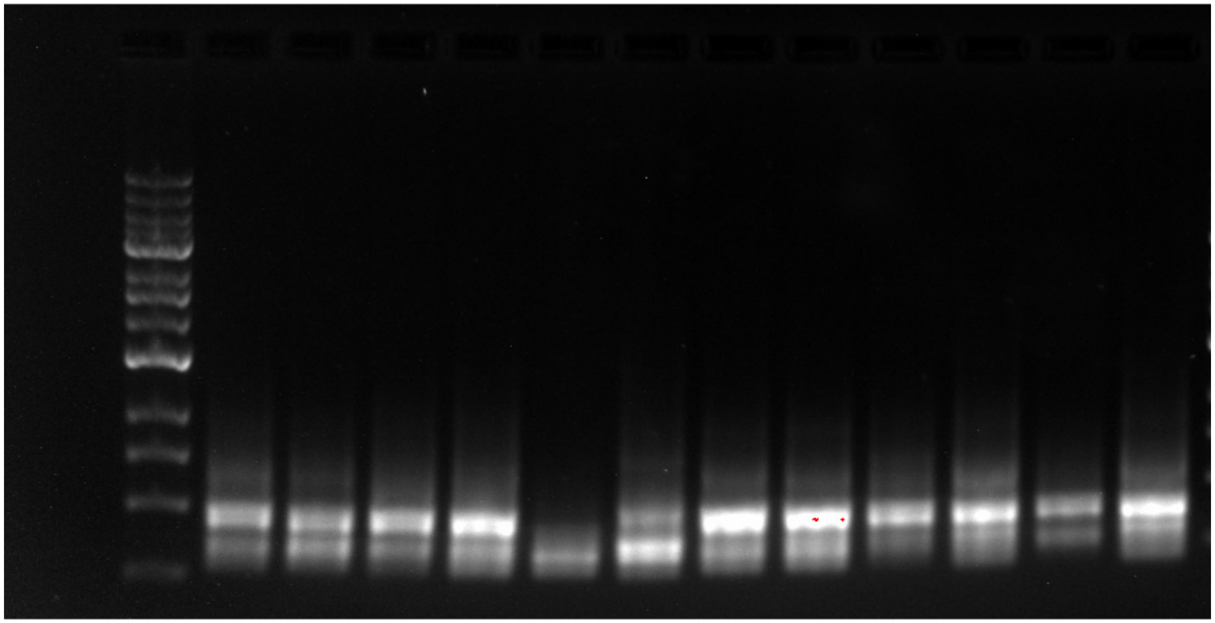

Primer:HB-9

**Band No**	**bp**	**Table S2 continued** ***Coffee*** **cultivars/populations**												
		2	3	4	5	6	7	8	9	10	11	12	13	**Band type**
1	200	1	0	1	1	0	0	1	1	1	1	1	1	P
2	250	0	1	0	1	0	0	0	0	0	0	0	0	P
3	300	0	1	1	0	1	0	1	1	1	1	1	1	P
	310	1	0	0	0	0	0	0	0	0	0	0	0	U
4	350	0	1	0	0	0	0	0	0	0	0	0	0	U
5	400	1	0	0	1	0	1	0	0	0	0	0	0	P
6	480	0	1	0	0	0	0	1	0	0	1	0	1	P
	580	1	1	1	1	0	1	1	0	1	1	1	1	P
7	730	0	0	0	0	0	1	0	0	0	0	0	0	U
	1000	0	0	0	0	0	0	0	0	0	1	0	1	P
**Tot. No. of bands**		**4**	**5**	**3**	**4**	**1**	**3**	**4**	**2**	**3**	**5**	**3**	**5**	

Image:

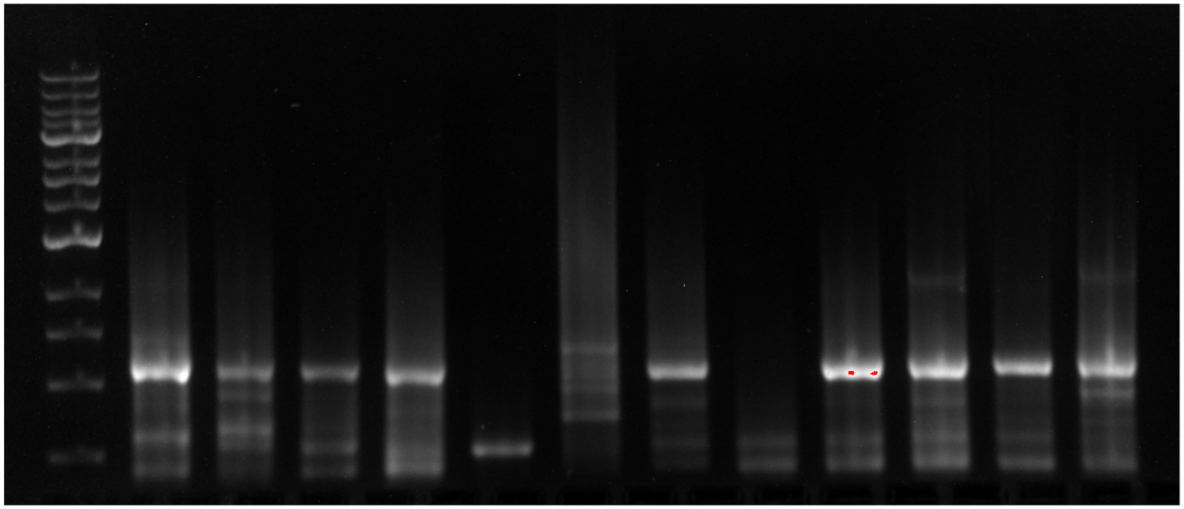

Primer Name:HB-10

**Band No**	**bp**	**Table S2 continued *Coffee* cultivars/populations**												
		2	3	4	5	6	7	8	9	10	11	12	13	**Band type**
1	250	1	1	1	1	0	1	1	1	1	1	1	1	P
2	275	0	0	1	1	0	0	1	1	1	1	1	1	P
3	300	1	1	1	1	1	1	1	1	1	1	1	1	M
	480	1	1	1	1	0	0	1	1	1	1	1	1	P
4	500	0	0	0	0	0	1	0	0	0	0	0	0	U
5	875	1	1	1	1	0	0	1	1	1	1	1	1	P
6	1200	1	1	1	1	0	1	0	0	0	0	0	1	P
7	2000	0	1	1	1	0	0	1	1	1	1	1	1	P
**Tot. No. of bands**		**5**	**6**	**7**	**7**	**1**	**4**	**6**	**6**	**6**	**6**	**6**	**7**	

Image:

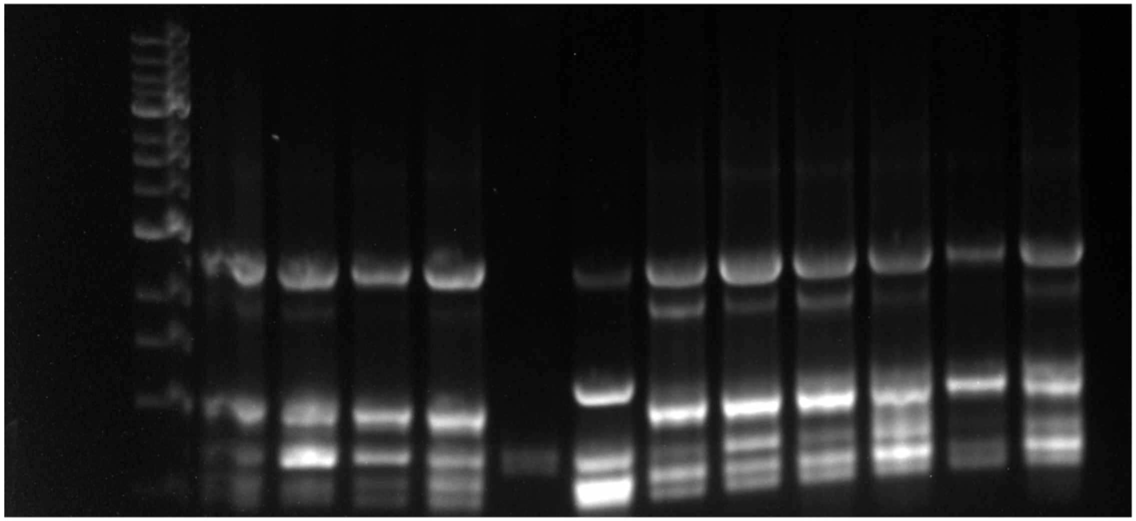

Primer Name:HB-12

**Band No**	**bp**	**Table S2 continued** *Coffee* **cultivars/populations**												
		2	3	4	5	6	7	8	9	10	11	12	13	**Band type**
1	270	1	1	1	1	1	1	1	1	1	1	1	1	M
2	400	1	1	1	1	0	1	1	1	1	1	1	1	P
3	500	1	1	1	1	0	0	1	0	1	1	0	0	P
4	625	1	1	1	1	1	1	1	1	1	1	1	1	M
5	800	1	1	0	0	1	0	1	0	0	1	0	0	P
6	1000	1	1	0	1	0	0	1	0	0	1	0	1	P
7	1500	0	0	0	0	1	0	0	0	0	0	0	0	U
**Tot. No. of bands**		**6**	**6**	**4**	**5**	**4**	**3**	**6**	**3**	**4**	**6**	**3**	**4**	

Image:

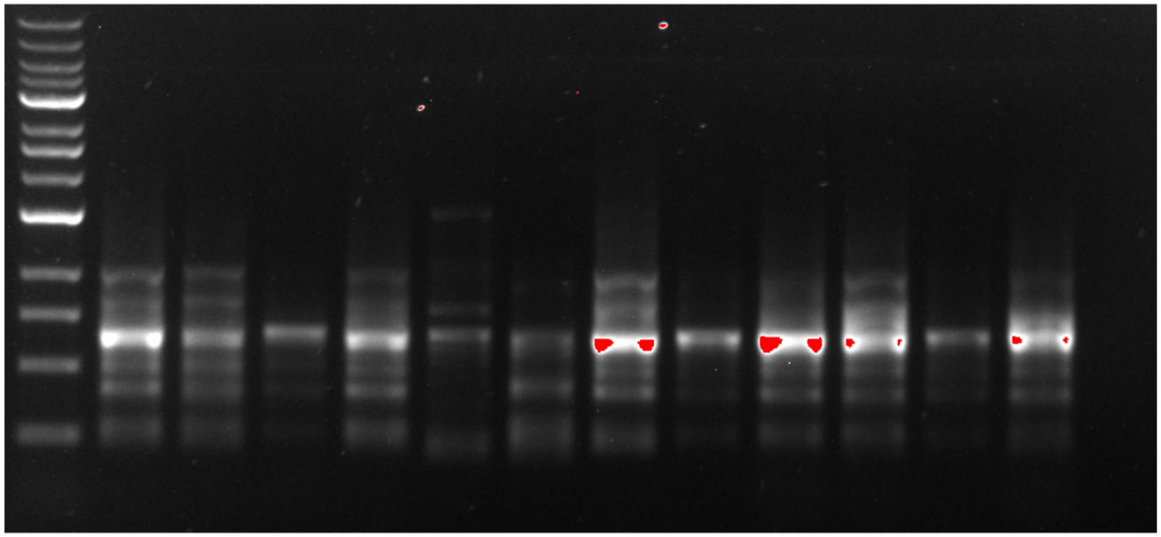

Primer Name:HB-13

**Band No**	**bp**	**Table S2 continued** *Coffee* **cultivars/populations**											
		2	3	4	5	6	7	8	9	10	11	12	13	**Band type**
1	300	1	1	1	1	1	1	1	1	1	1	1	1	M
2	489	1	1	1	1	1	0	1	1	1	1	1	1	P
3	650	1	1	1	1	0	0	1	1	1	1	0	1	P
4	800	1	1	1	1	0	0	1	1	1	1	0	1	P
5	1000	1	1	1	1	0	0	1	1	1	1	0	1	P
	1200	0	0	0	0	1	0	0	0	0	0	1	1	P
**Tot. No. of bands**		**5**	**5**	**5**	**5**	**3**	**1**	**5**	**5**	**5**	**5**	**3**	**6**	

Image:

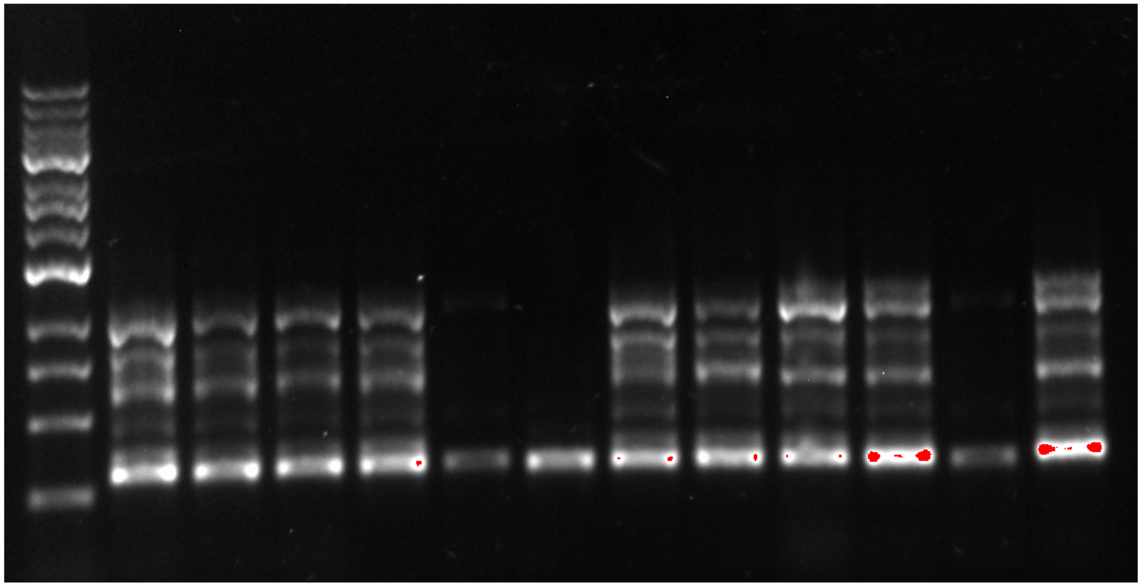

Primer Name:HB-14

**Band No**	**bp**	**Table S2 continued** *Coffee* **cultivars/populations**												
		2	3	4	5	6	7	8	9	10	11	12	13	**Band type**
1	400	0	0	0	0	0	0	1	0	0	1	0	1	P
2	500	1	0	0	0	0	0	1	1	1	1	1	1	P
3	750	1	1	1	1	1	1	1	1	1	1	1	1	M
4	1000	1	0	0	0	1	1	1	1	1	1	1	1	P
5	1300	1	1	0	0	1	0	1	1	1	1	1	1	P
6	2250	1	0	0	0	0	0	1	1	1	1	1	1	P
7														
**Tot. No. of bands**		**5**	**2**	**1**	**1**	**3**	**2**	**6**	**5**	**5**	**6**	**5**	**6**	

Image:

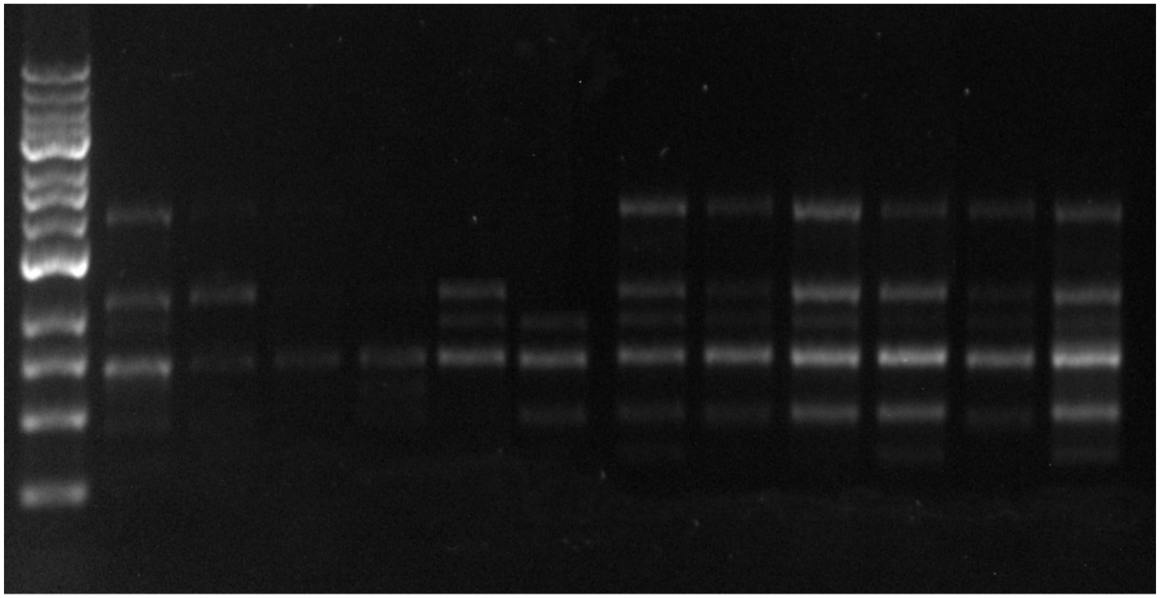

Primer Name:814

Band No	bp	**Table S2 continued** *Coffee* **cultivars/populations**												
		2	3	4	5	6	7	8	9	10	11	12	13	**Band type**
1	580	1	0	1	0	0	0	0	0	0	0	0	0	P
2	625	1	0	1	1	0	0	1	1	0	0	1	1	P
3	700	1	1	1	1	1	1	1	1	1	1	1	1	M
4	900	1	1	1	1	0	1	1	1	1	1	1	1	P
5	1100	1	1	1	1	1	1	1	1	1	1	1	1	M
6	1300	1	1	1	1	1	1	1	1	0	1	1	1	P
	1500	0	1	0	0	1	1	0	0	0	1	0	1	P
**Tot. No. of bands**		**6**	**5**	**6**	**5**	**4**	**5**	**5**	**5**	**3**	**5**	**5**	**6**	

Image:

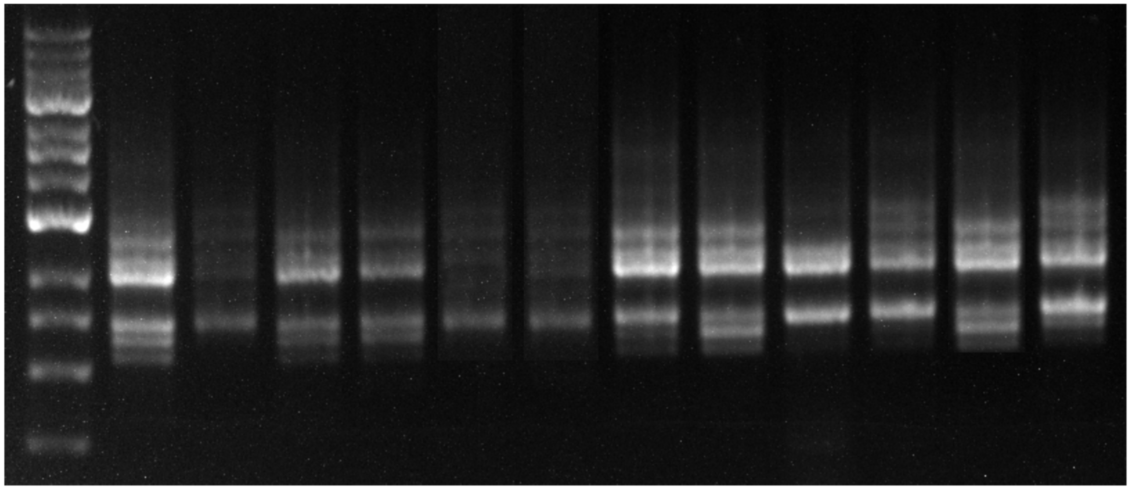

Primer Name:826

Band No	bp	Table S2 continued *Coffee* cultivars/populations												
		2	3	4	5	6	7	8	9	10	11	12	13	**Band type**
1	325	1	1	1	1	1	1	1	1	1	1	1	1	M
2	400	1	1	1	0	0	0	1	1	1	1	1	1	P
3	550	1	1	1	0	0	0	1	1	1	1	1	1	P
4	700	1	1	1	0	0	0	1	1	1	1	1	1	P
5	800	0	0	1	0	0	1	0	0	1	1	0	1	P
6	1000	1	1	0	0	0	0	1	1	1	1	1	1	P
**Tot. No. of bands**		**5**	**5**	**5**	**1**	**1**	**2**	**5**	**5**	**6**	**6**	**5**	**6**	

Image:

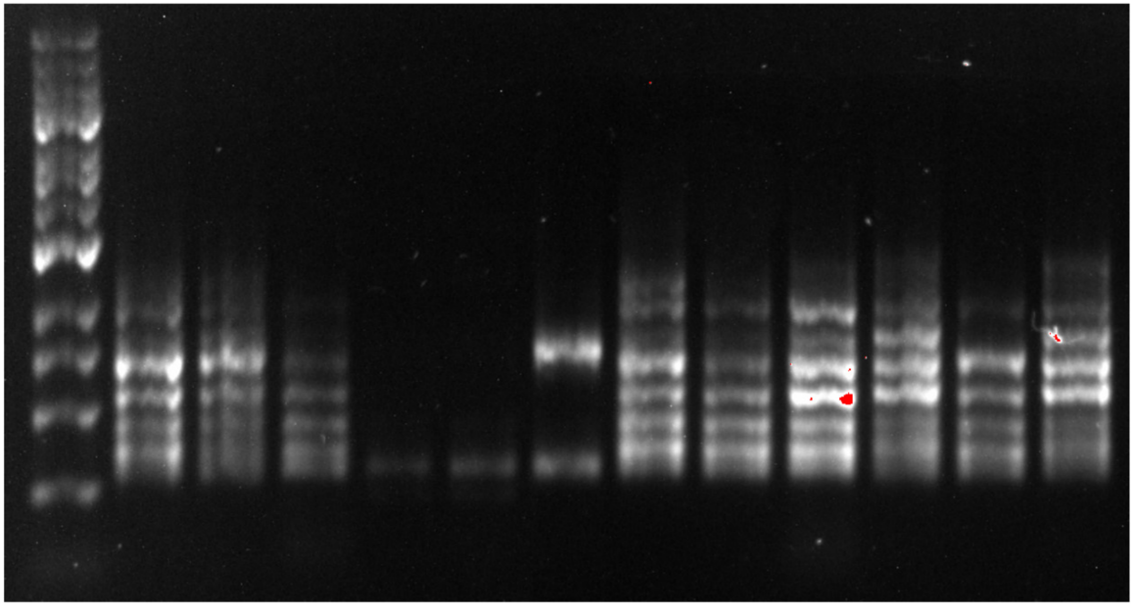

Primer Name: SCOT3

**Band No**	**bp**	**Table S2 continued *Coffee* cultivars/populations**												
		2	3	4	5	6	7	8	9	10	11	12	13	**Band type**
1	100	0	1	0	1	0	0	0	1	1	1	1	0	P
2	280	0	0	0	0	1	1	0	1	0	0	0	0	P
3	300	1	0	1	1	0	0	0	1	1	0	0	0	P
4	450	1	0	0	0	0	0	0	0	0	0	0	0	U
5	550	1	0	0	1	1	1	0	0	0	0	0	1	P
6	650	0	0	0	0	0	0	0	0	1	0	0	0	U
7	750	1	0	0	1	0	0	1	1	1	1	1	1	P
8	800	0	1	1	0	0	0	0	0	0	0	0	0	P
**Tot. No. of bands**		**4**	**2**	**2**	**4**	**2**	**2**	**1**	**4**	**4**	**2**	**2**	**2**	

Image:

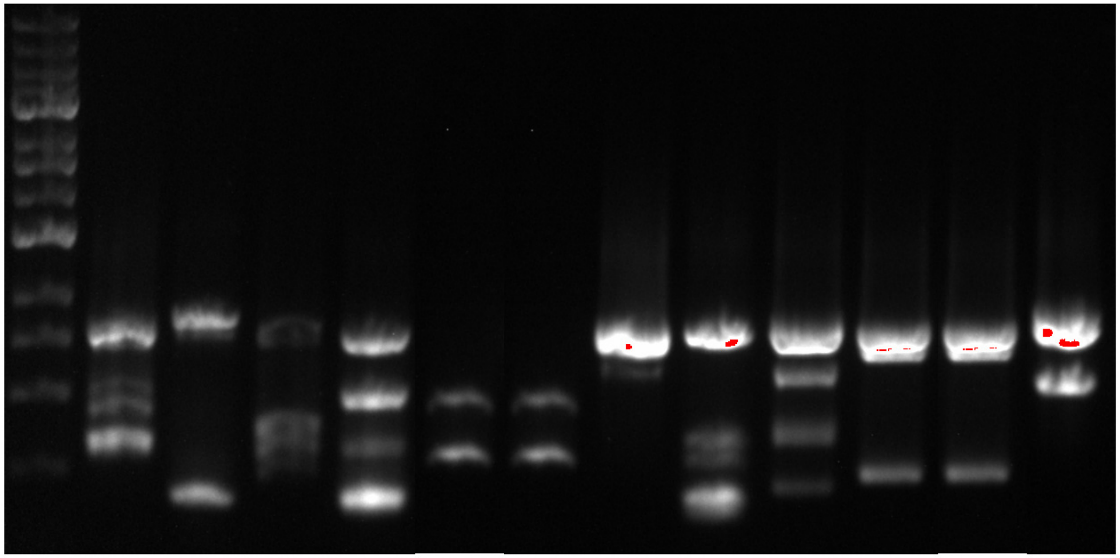

Primer Name:SCOT4

**Band No**	**bp**	**Table S2 continued *Coffee* cultivars/populations**												
		2	3	4	5	6	7	8	9	10	11	12	13	**Band type**
1.	230	1	0	0	1	0	1	1	1	1	1	1	1	P
2.	270	1	1	1	0	1	1	1	1	1	1	1	1	P
3.	400	0	1	0	0	0	0	0	0	1	1	1	1	P
4.	600	0	1	1	0	0	0	0	0	1	0	1	0	P
5.	750	1	0	0	1	0	0	1	1	1	1	1	1	P
6.	850	1	1	1	1	0	1	1	1	1	1	1	1	P
**Tot. No. of bands**		**4**	**3**	**3**	**3**	**1**	**3**	**4**	**4**	**6**	**5**	**6**	**5**	

Image:

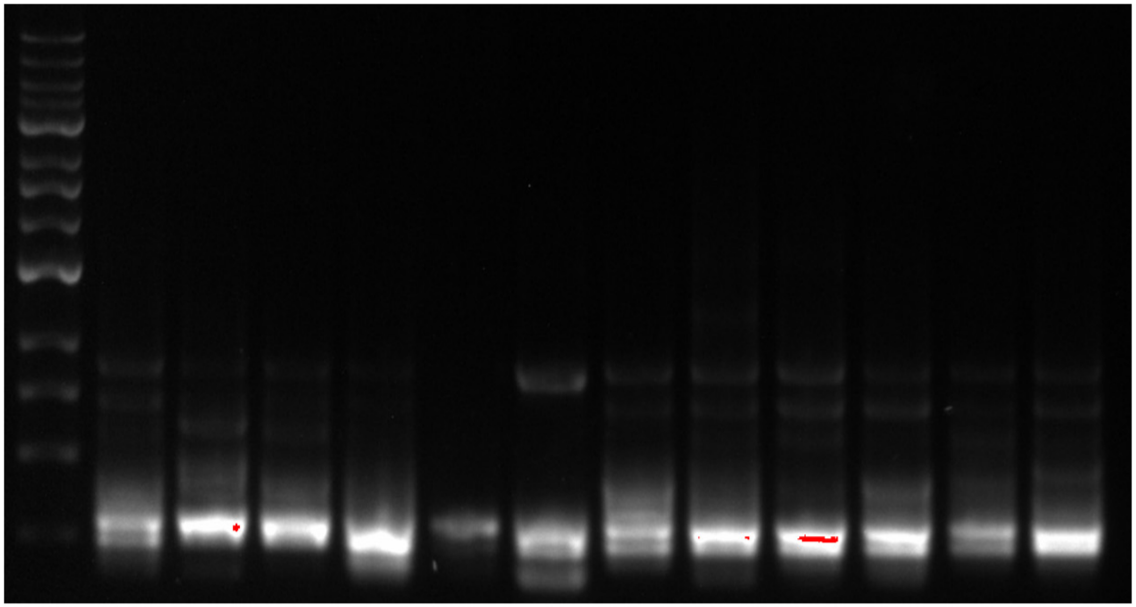

Primer Name:SCOT7

Band No	bp	**Table S2 continued** *Coffee* **cultivars/populations**											
		2	3	4	5	6	7	8	9	10	11	12	13	**Band type**
1.	280	0	0	0	1	0	1	0	1	1	0	0	0	P
2.	300	1	1	1	0	1	0	1	0	0	1	1	1	P
3.	400	1	0	0	1	0	1	0	0	0	0	1	1	P
4.	430	0	0	1	0	1	0	1	1	0	0	0	1	P
5.	450	0	0	0	0	0	0	1	0	1	0	0	0	P
6.	550	1	0	0	1	0	0	1	0	0	0	1	1	P
7.	650	1	1	1	1	1	1	1	1	1	1	1	1	M
8.	760	0	1	0	1	0	1	0	1	0	1	0	0	P
9.	825	1	1	0	1	0	0	1	0	1	0	1	1	P
10.	1200	0	0	0	0	0	1	0	0	0	0	0	0	U
**Tot. No. of bands**		**5**	**4**	**3**	**6**	**3**	**5**	**6**	**4**	**4**	**3**	**5**	**6**	

Image:

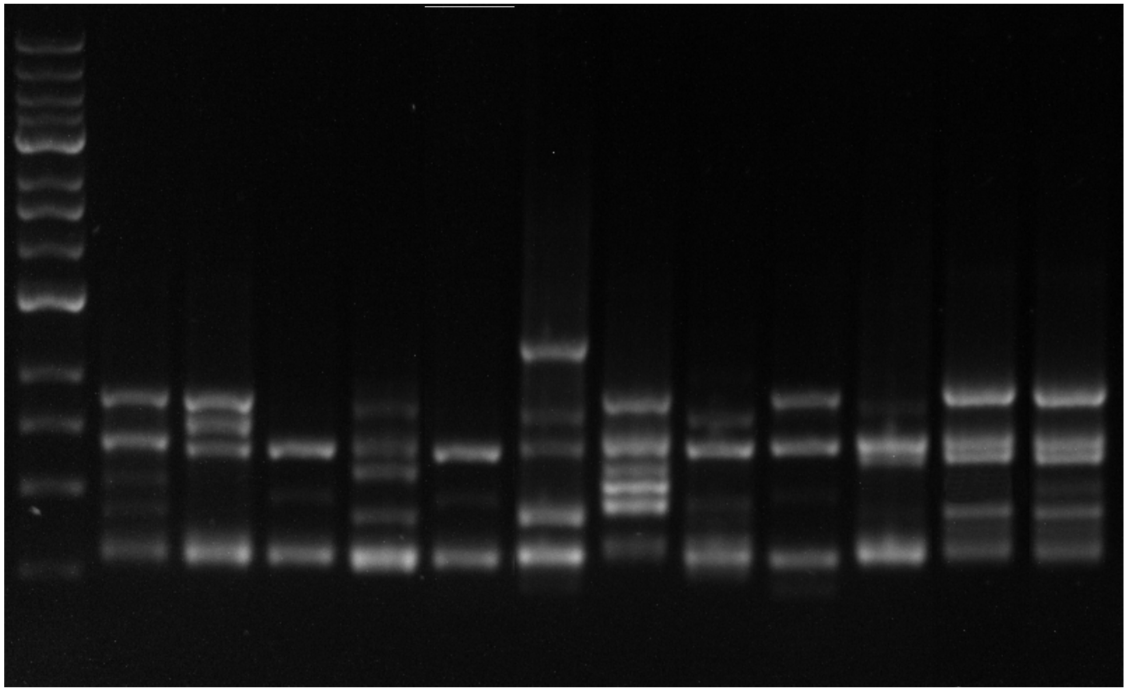

Primer Name:SCOT8

**Band No**	**bp**	**Table S2 continued *Coffee* cultivars/populations**												
		2	3	4	5	6	7	8	9	10	11	12	13	**Band type**
1.	400	1	1	1	1	1	0	1	1	1	1	1	1	P
2.	500	1	1	1	1	1	1	1	1	1	1	1	1	M
3.	700	1	1	1	1	1	1	1	1	1	1	1	1	M
4.	900	0	0	0	0	0	0	0	0	1	0	0	0	U
5.	1000	1	1	1	1	0	0	1	1	0	1	1	1	P
6.	1300	0	0	0	0	0	0	0	0	1	0	0	0	U
7.	1400	0	0	0	0	0	1	1	0	0	0	1	0	P
8.	1500	0	1	0	1	0	0	0	0	0	0	0	0	U
9.	1750	1	1	1	1	0	0	1	1	1	1	0	1	P
**Tot. No. of bands**		**5**	**6**	**5**	**6**	**3**	**3**	**6**	**5**	**6**	**5**	**5**	**5**	

Image:

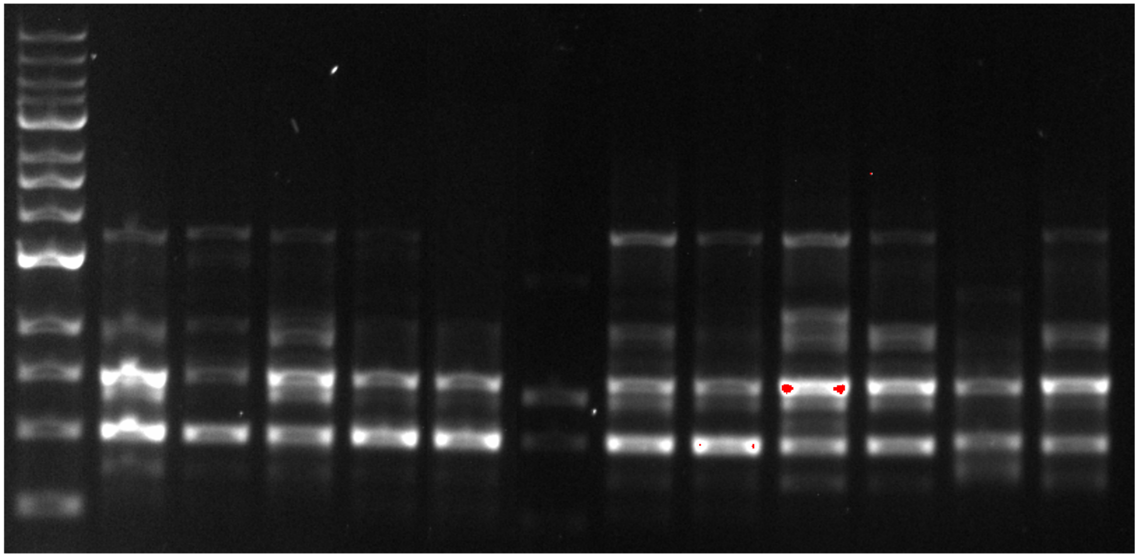

Primer Name:SCOT9

**Band No**	**bp**	**Table S2 continued** *Coffee* **cultivars/populations**											
		2	3	4	5	6	7	8	9	10	11	12	13	**Band type**
1.	750	1	1	1	1	1	1	1	1	1	1	0	1	P
2.	800	1	0	0	0	0	1	1	1	0	0	0	1	P
3.	1000	1	0	0	0	0	1	1	0	0	0	1	0	P
4.	1200	1	1	1	1	1	1	1	1	1	1	1	1	M
5.	1500	1	0	1	1	0	1	1	1	1	1	1	1	P
6.	2250	1	1	1	1	1	1	1	1	1	1	1	1	M
**Tot. No. of bands**		**6**	**3**	**4**	**4**	**3**	**6**	**6**	**5**	**4**	**4**	**4**	**5**	

Image:

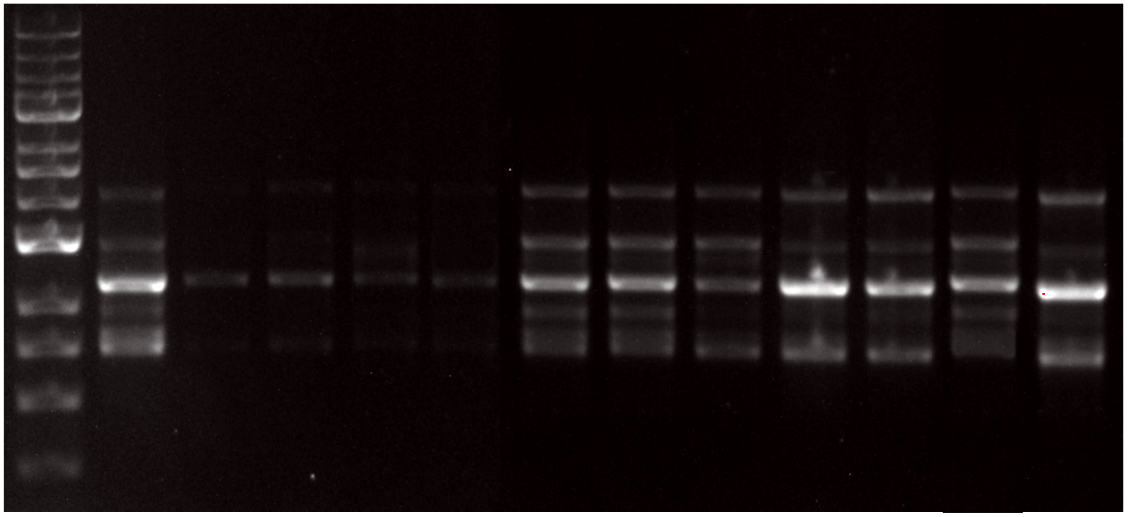

Primer Name:SCOT13

Band No	bp	Table S2 continued *Coffee* cultivars/populations											
		2	3	4	5	6	7	8	9	10	11	12	13	**Band type**
1.	450	1	0	0	0	0	0	0	0	0	0	0	0	U
2.	650	1	0	0	0	0	0	0	0	0	0	0	0	U
3.	750	1	0	0	1	1	0	0	0	1	0	1	1	P
4.	850	1	1	0	1	1	0	1	0	1	0	1	0	P
5.	950	0	0	0	0	0	0	0	0	0	0	0	1	U
6.	1000	0	0	0	0	0	0	0	1	0	0	1	0	P
7.	1100	0	0	0	0	0	0	0	1	0	0	0	0	U
8.	1200	1	0	0	0	0	0	0	0	1	0	0	1	P
9.	1750	1	0	1	1	1	1	1	1	1	1	1	1	M
10.														
**Tot. No. of bands**		**6**	**1**	**1**	**3**	**3**	**1**	**2**	**3**	**4**	**1**	**4**	**4**	

Image:

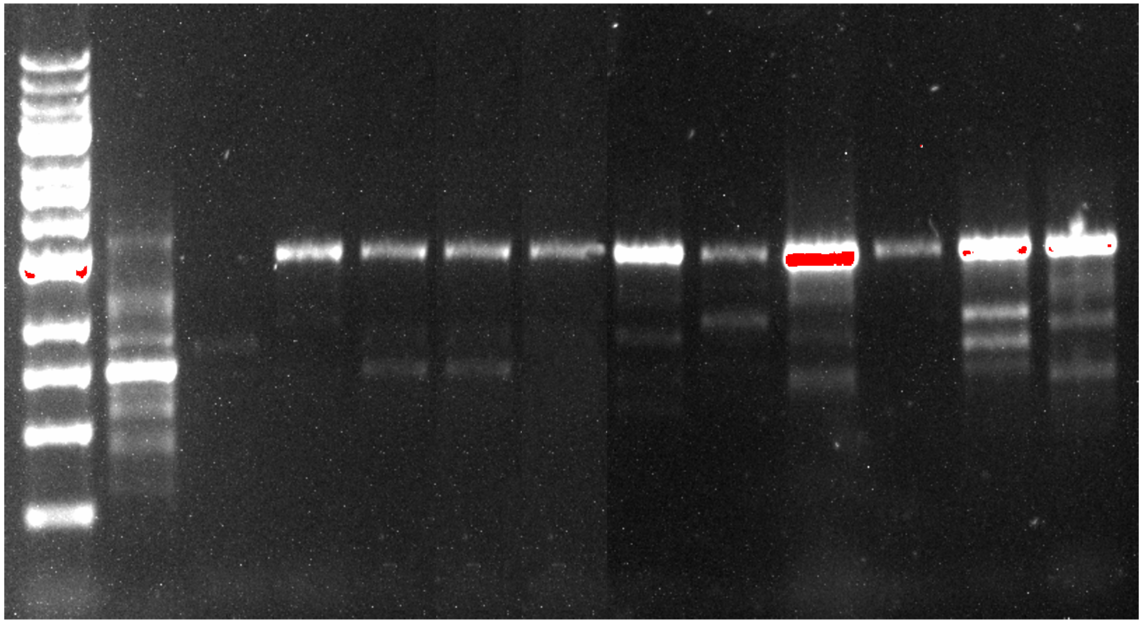

Primer Name:SCOT14

Band No	bp	Table S2 continued *Coffee* cultivars/populations											
		2	3	4	5	6	7	8	9	10	11	12	13	**Band type**
1.	250	0	0	0	0	0	1	0	0	0	0	0	0	U
2.	400	1	0	1	1	0	0	1	1	1	1	1	1	P
3.	450	1	0	0	0	0	0	0	0	0	0	0	0	U
4.	500	0	1	1	0	0	1	1	1	1	1	1	1	P
5.	530	0	0	0	0	1	1	0	0	0	0	0	0	P
6.	550	1	0	1	1	0	0	1	1	1	1	1	1	P
7.	600	0	1	0	0	0	0	0	0	0	0	0	0	U
8.	700	0	0	0	1	0	1	1	1	1	1	0	1	P
9.	850	0	0	0	1	0	1	1	0	0	0	0	0	P
10.	950	0	1	1	1	0	0	1	1	1	1	0	1	P
11.	1100	1	0	1	1	1	1	1	1	1	1	0	1	P
12.	1300	0	1	0	0	0	0	0	0	0	1	0	0	P
**Tot. No. of bands**		**4**	**4**	**5**	**6**	**2**	**6**	**7**	**6**	**6**	**7**	**3**	**6**	

Image:

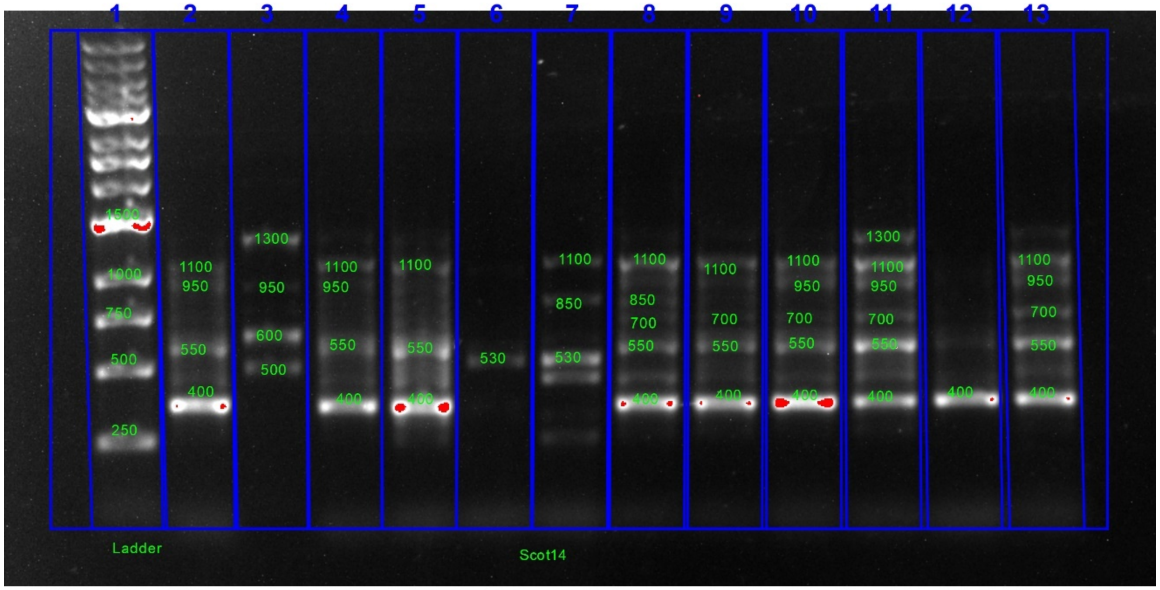

1 2 3 4 5 6 7 8 9 10 11 12 13
1500
1000
750
500
250
Ladder
Scot14

Primer Name:SCOT15

Band No	bp	**Table S2 continued**					**_Coffee_ cultivars/populations**						
		2	3	4	5	6	7	8	9	10	11	12	13	**Band type**
1.	400	1	1	1	1	1	1	1	1	1	1	0	1	P
2.	500	0	0	0	0	0	1	0	0	0	0	0	0	U
3.	550	0	0	1	1	1	1	0	1	1	1	1	1	P
4.	580	0	0	0	0	0	0	1	1	0	0	0	0	P
5.	600	1	1	1	1	1	1	0	0	0	1	0	1	P
6.	750	0	0	0	0	0	0	1	1	1	0	1	0	P
7.	950	0	0	0	0	0	0	1	1	0	0	0	0	P
8.	1200	1	1	1	1	1	0	1	1	0	1	0	1	P
9.	1250	1	1	1	1	1	1	1	1	0	1	0	1	P
10.	1500	0	1	0	0	0	0	0	0	0	0	0	0	U
**Tot. No. of bands**		**4**	**5**	**5**	**5**	**5**	**5**	**6**	**7**	**3**	**5**	**2**	**5**	

Image:

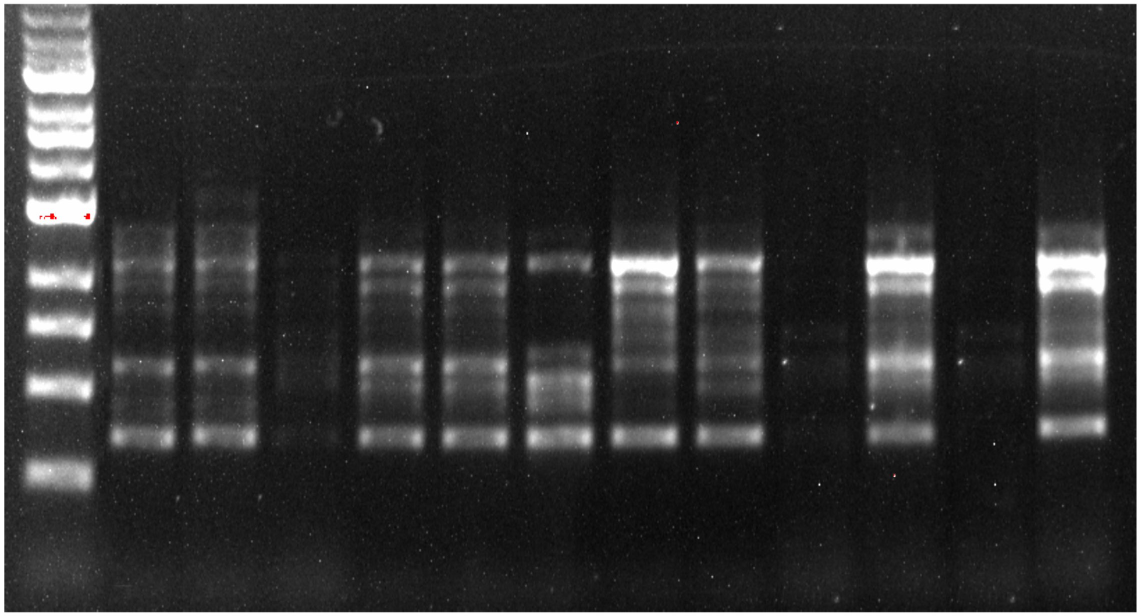

Primer Name: SCOT16

Band No	bp	**Table S2 continued** *Coffee* **cultivars/populations**											
		2	3	4	5	6	7	8	9	10	11	12	13	**Band type**
1.	450	1	0	0	0	0	0	0	0	0	1	1	1	P
2.	550	1	1	1	1	1	1	1	1	1	1	1	1	M
3.	600	1	1	1	1	1	0	1	1	1	1	1	1	P
4.	650	1	1	1	1	1	0	1	1	1	1	1	1	P
5.	750	0	0	1	0	0	0	1	1	1	1	0	1	P
6.	800	0	1	0	0	0	0	0	0	0	0	0	0	U
7.	850	1	0	1	1	1	0	1	1	1	1	0	1	P
8.	1100	1	1	0	0	0	0	0	0	0	0	0	0	P
9.	1250	1	0	1	1	1	0	1	1	1	1	0	1	P
**Tot. No. of bands**		**7**	**5**	**6**	**5**	**5**	**1**	**6**	**6**	**6**	**7**	**4**	**7**	

Image:

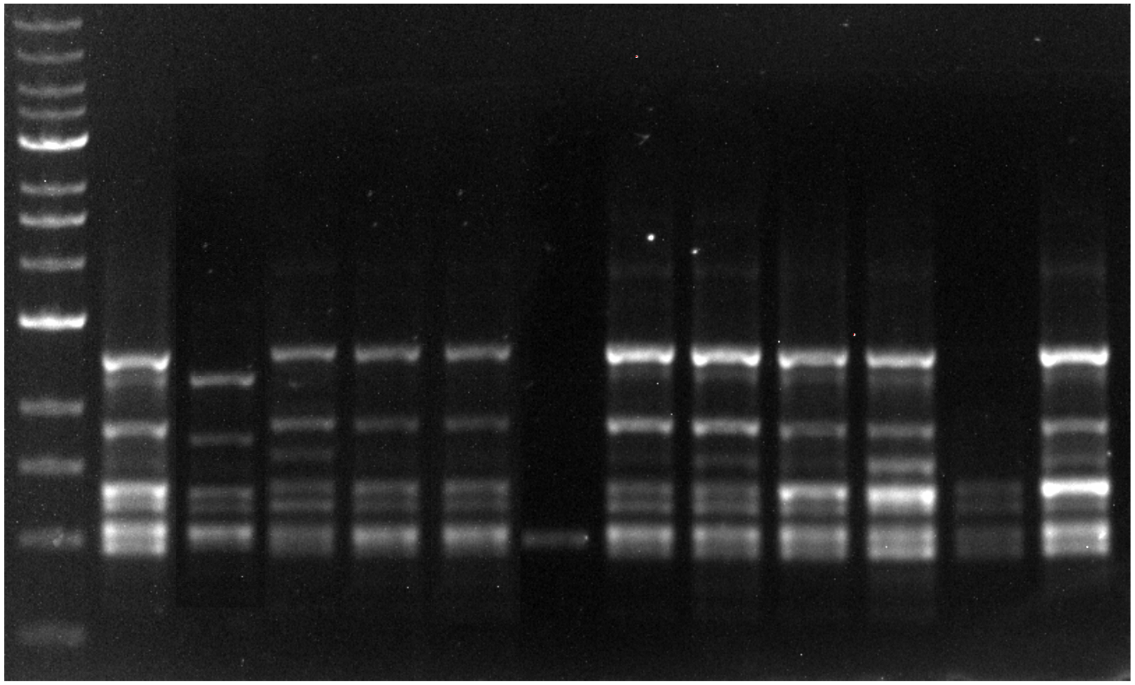

Table S3. List of the examined morphological traits, their descriptor states, and given codes for data analysis.

Serial	Character	Descriptor State	Code
**1**	Plant Shape	Conical	0
		Ellipsoid	1
		Pyramidal	2
**2**	Growth Habit	Shrub	0
		Tree	1
**3**	Plant Height	Short < 3 M	0
		Intermediate (3-5) M	1
		Long > 5 M	2
**4**	Canopy Dm	Narrow < 3 M	0
		Intermediate (3-4) M	1
		Wide > 4 M	2
**5**	Internode Length on Primary Branch	Short < 7 CM	0
		Intermediate (7-10) CM	1
		Long > 10 CM	2
**6**	Leaf Length	Short < 10 CM	0
		Intermediate (10-13) M	1
		Long > 13 CM	2
**7**	Leaf Width	Narrow < 5 CM	0
		Intermediate (5-7) CM	1
		Wide > 7 CM	2
**8**	Leaf Shape	Lanceolate	0
		Ovate	1
		Elliptic	2

**9**	Young Leaf Anthocyanin Colouration	Week or absent<br>Strong	0<br>1
**10**	Leaf Margin Undulation	Week or absent<br>Intermediate<br>Strong	0<br>1<br>2
**11**	Leaf Petiole Length Cm	Short < 5<br>Intermediate (5:6)<br>Long > 6	0<br>1<br>2
**12**	Leaf Petiole Colour	Dark green<br>Green	0<br>1
**13**	No. of Flowers	small< 15<br>Intermediate 15:20<br>high > 20	0<br>1<br>2
**14**	Inflorescence Position	Axillary<br>Terminal	0<br>1
**15**	Inflorescence on Old Wood	Absent<br>Present	0<br>1
**16**	No. of Flower Fascicles	small< 8<br>Intermediate 8:10<br>high > 10	0<br>1<br>2
**17**	Fruit Size	small< 1<br>Intermediate 1:1.5<br>high > 1.5	0<br>1<br>2<br>3
**18**	Fruit Shape	Elliptic<br>Circular	0<br>1

**19**	Fruit Thickness	Thin< 3mm<br>Intermediate 3:5 mm<br>Thick > 5	0<br>1<br>2
**20**	Fruit adherence to branch	Week<br>Strong	0<br>1
**21**	Cherry Colour	Orange<br>Light red	0<br>1
**22**	Seed width	Narrow< 5mm<br>Intermediate 5:10 mm<br>Broad > 10 mm	0<br>1<br>2
**23**	100 Fruit fresh weight	small< 150<br>Intermediate 150:200<br>high > 200	0<br>1
**24**	100 Fruit dray weight	small< 45<br>Intermediate 45:50<br>high > 50	0<br>1<br>2
**25**	100 Seed dray weight	small< 25<br>Intermediate 25:30<br>high > 30	0<br>1<br>2

Table S4. Shows Principal Component Analysis (PCA) results among the examined coffee arabica accessions.

	**PC 1**	**PC 2**	**PC 3**	**PC 4**	**PC 5**	**PC 6**	**PC 7**	**PC 8**	**PC 9**	**PC 10**	**PC 11**
**R4**	-0.9963	3.5319	1.5352	-1.9519	-1.1244	-0.14268	-1.1029	1.9751	-1.064	0.36911	1.3898
**Y3**	-0.27751	-2.7472	3.3428	2.0115	0.9922	-0.74446	-1.5301	1.4099	0.0024	0.66292	-0.9578
**Y5**	1.7917	-2.7824	0.3489	-1.0894	1.1432	1.5212	-0.0371	- 0.2904	-1.375	-2.435	1.1316
**R8**	-1.2075	0.8457	0.3058	-3.1245	2.9306	0.0440	1.6189	0.267	1.2077	0.0507	-1.401

**R9**	-3.0921	-3.033	-0.7295	-1.0878	-2.4437	-2.349	1.7249	-0.321	-0.765	0.3105	0.1006
**R111**	-5.5843	0.2872	-1.3443	1.6744	0.3322	2.7259	-0.766	-0.541	0.231	0.113	0.191
**R112**	0.88171	3.0438	0.6634	2.0716	0.2394	-0.534	1.5164	-1.4788	-2.3669	-0.36894	-1.1082
**R113**	2.166	0.171	-2.52	1.9185	-0.387	0.270	1.662	2.991	0.7497	-0.344	-0.0373
**Y6**	2.7895	-1.0481	-2.0902	-0.701	1.1877	0.38416	-1.0138	-0.819	-1.0342	2.7368	0.4214
**R114**	0.8827	0.679	1.5506	1.3056	0.469	-0.844	1.253	-1.581	2.261	0.3158	2.0408
**Y2**	0.269	1.063	-2.041	-0.018	0.1050	-2.508	-2.961	-0.718	0.937	-1.462	-0.506
**Y7**	2.3757	-0.0128	0.984	-1.0072	-3.443	2.1777	-0.364	-0.891	1.215	0.051	-1.264
